# Collaborative team dynamics and scholarly outcomes of multidisciplinary research teams: A mixed-methods approach

**DOI:** 10.1017/cts.2023.9

**Published:** 2023-02-03

**Authors:** Emily Slade, Philip A. Kern, Robert L. Kegebein, Chang Liu, Joel C. Thompson, Thomas H. Kelly, Victoria L. King, Robert S. DiPaola, Hilary L. Surratt

**Affiliations:** 1 Department of Biostatistics, University of Kentucky, Lexington, KY, USA; 2 Department of Internal Medicine, University of Kentucky, Lexington, KY, USA; 3 Center for Clinical and Translational Science, University of Kentucky, Lexington, KY, USA; 4 Markey Cancer Center, University of Kentucky, Lexington, KY, USA; 5 Department of Behavioral Science, University of Kentucky, Lexington, KY, USA; 6 Office of the Provost, University of Kentucky, Lexington, KY, USA

**Keywords:** Team science, transdisciplinary research, collaboration, mixed methods, evaluation

## Abstract

**Introduction:**

Impactful, transdisciplinary scientific discoveries are created by teams of researchers spanning multiple disciplines, but collaboration across disciplines can be challenging. We examined how team dynamics and collaboration are related to successes and barriers faced by teams of researchers from multiple disciplines.

**Methods:**

A mixed-methods approach was used to examine 12 research teams granted multidisciplinary pilot awards. Team members were surveyed to assess their team dynamics and individual views about transdisciplinary research. Forty-seven researchers (59.5%) responded, including two to eight members from each funded team. Associations were examined between collaborative dynamics and scholarly product outcomes, including manuscripts, grant proposals, and awarded grants. One member from each team was selected for an in-depth interview to contextualize and extend information about collaborative processes, successes, and barriers to performing transdisciplinary research.

**Results:**

Quality of team interactions was positively associated with achievement of scholarly products (*r* = 0.64, *p* = 0.02). Satisfaction with team members (*r* = 0.38) and team collaboration scores (*r* = 0.43) also demonstrated positive associations with achievement of scholarly products, but these were not statistically significant. Qualitative results support these findings and add further insight into aspects of the collaborative process that were particularly important to foster success on multidisciplinary teams. Beyond scholarly metrics, additional successes from the multidisciplinary teams were identified through the qualitative portion of the study including career development and acceleration for early career researchers.

**Conclusions:**

Both the quantitative and qualitative study results indicate that effective collaboration is critical to multidisciplinary research team success. Development and/or promotion of team science-based trainings for researchers would promote these collaborative skills.

## Introduction

Multidisciplinary teams of researchers are needed to promote innovative transdisciplinary research approaches to recalcitrant health problems, especially those that aim to generate actionable insights for clinical care or public health [1,2]. For the purpose of this work, “multidisciplinary” refers to the existence of multiple distinct disciplines, and “transdisciplinary” refers to the integration of ideas from multiple disciplines to create insights that transcend the distinct fields [3]. “Team science” refers to the act of investigators with differing expertise (multidisciplinary teams) working together to integrate ideas from different domain areas to answer a biomedical question (transdisciplinary insight) [4]. Past work studying the science of team science has suggested that multidisciplinary teams publish more and produce more innovative work than individual investigators or teams of investigators from the same domain area [5–9].

Despite the importance of team science in creating impactful biomedical research, it has been noted that working collaboratively often requires more time and resources than working alone [10–12]. There may also be increased difficulty in communicating with collaborators who work in different domain areas [13,14]. For example, communication is particularly challenging when researchers’ areas of expertise span different stages of the translational science research spectrum as these stages typically take fundamentally different approaches to scientific discovery, such as small, well-controlled basic science versus large, population-level data science [15]. Organizational barriers may also exist for multidisciplinary research teams such as a lack of shared physical space for collaborators across disciplines [16], difficulty in accounting for collaborative work in individual performance metrics [17], or a lack of technological resources to facilitate multidisciplinary collaboration [12,18].

Institutions may seek to reduce barriers for multidisciplinary research teams through various avenues such as hosting research networking events to introduce researchers across disciplines or providing pilot funding mechanisms that specifically incentivize transdisciplinary work. As with any pilot funding mechanism, the goal of the institution is typically to receive return on their investment by generating research momentum for a multidisciplinary team that leads to clinical breakthroughs and subsequent external funding to continue the work. For this reason, subsequent research funding as well as bibliographic measures (i.e., publications) are commonly used as metrics to evaluate team performance on pilot awards [2]. Despite their popularity, these scholarly metrics are limited in their ability to capture information about collaborative team performance, and thus, many have recommended the use of a mixed-methods approach to contextualize and extend the information provided by counts of publications and grants [19].

At our institution, the University of Kentucky, two pilot awards that specifically incentivize research from multidisciplinary teams were developed – the Multidisciplinary Value Program (MVP) and the Value of Innovation to Implementation Program (VI^2^P). The overall goal of this study is to measure the scholarly outcomes from research teams funded by these two programs and to examine the association between collaborative team dynamics and research outcomes. We take a mixed-methods approach to provide objective, quantitative data about team experiences and outcomes as well as to contextualize this information and provide additional insight into team dynamics that is not easily captured by traditional quantitative measures alone.

## Methods

### Study Overview and Participants

Eligible participants were members on the 12 multidisciplinary research teams that received pilot funding through the MVP or VI^2^P pilot award programs from 2016 to 2018. These programs required teams to consist of at least one physician investigator and at least one investigator with a strong prior record of research funding. There was no requirement related to history of prior collaboration between team members. The funding mechanisms emphasized collaborative team science projects that aimed to implement evidence-based strategies to address a health challenge faced in Kentucky. Awards were provided for up to $110,000 in total direct costs over an 18-month period. For more information about these pilot award programs, see Surratt et al. [20].

All named members on the pilot awards (79 total individuals) were asked to participate in a survey to assess team dynamics, collaboration, and research outcomes. The survey was developed on Qualtrics and distributed to eligible participants via email. Fifty (63.3%) of the eligible participants started the survey, and 47 (59.5%) of the responses were deemed usable for analysis. In this mixed-methods study, follow-up interviews were conducted with one member of each of the 12 pilot award teams to contextualize the survey findings. All 12 interviewees were either the PI, MPI, or Co-I on their respective pilot awards. These semi-structured, in-depth interviews were organized by an interview guide containing broad questions on key topical areas, including: team formation and composition, team building and strategies for working as a team, barriers and supports for team science at the institution, as well as transdisciplinary impacts of the pilot awards. Institutional Review Board approval for the study was obtained from the University of Kentucky Medical IRB.

### Survey Measures

In total, eight independent variables were assessed from the survey responses. Three of these independent variables represent interactions among the pilot team members, including: (1) satisfaction with team members, (2) assessment of team collaboration, and (3) quality of team interactions. Two independent variables assess general views about transdisciplinary research outside of the interactions on the pilot team, including: (1) individual attitude about transdisciplinary research and (2) individual assessment of importance of collaboration at their institution. Last, three independent variables represent measures related to how long the team members have been collaborating with each other, including: (1) additive team tenure, (2) collective team tenure, and (3) dispersion in team tenure. All eight of these independent variables were assessed via individual survey responses and also aggregated at the team level as the median of individual responses among members of each team. The survey items were adapted from preexisting team science evaluation instruments as follows.

#### Satisfaction with team members

Participants were asked to rate their level of satisfaction with each of their pilot team collaborators on a 5-point scale from *not at all satisfied* to *completely satisfied*. This scale was extracted from the National Cancer Institute’s Transdisciplinary Research on Energetics and Cancer (TREC) Baseline Researcher Survey [10]. Each participant’s ratings of their collaborators were averaged to calculate their overall satisfaction with pilot team members.

#### Assessment of team collaboration

To assess the interpersonal collaborative processes and collaborative productivity on the pilot award project, we used the eight items from the “Collaboration” section on the TREC Baseline Researcher Survey [10]. Each item was assessed using a 5-point response scale from *strongly disagree* to *strongly agree* or from *very poor* to *excellent*. Scores on each of these eight items were averaged to calculate each participant’s overall assessment of team collaboration.

#### Quality of team interactions

To measure the quality of pilot team interactions, we used the Team Performance Scale (TPS) which asks participants to evaluate their overall experience with their teams using 18 items [21]. Each item has a 6-point response scale as *never*, *very rarely*, *rarely*, *occasionally*, *very frequently*, and *always*. Ratings from these 18 items were averaged to calculate each participant’s overall assessment of the quality of their pilot team interactions.

#### Attitude about transdisciplinary research

Participants were asked to assess their general attitude about transdisciplinary research outside of the pilot project using the 15 items from the “Transdisciplinary Research” section of the Transdisciplinary Tobacco Use Research Centers (TTURC) Researcher Form [22]. Each item was assessed using a 5-point response scale from *strongly disagree* to *strongly agree*. Scores on each of these 15 items were averaged to calculate each participant’s overall attitude about transdisciplinary research in general.

#### Assessment of importance of collaboration at the institution

Participants were asked to assess the importance of collaboration at the home institution using four items from the “Support and Recognition” section of the TTURC Researcher Form [22]. Each item was assessed using a 5-point response scale from *not at all important* to *extremely important*. Scores on each of these four items were averaged to calculate each participant’s overall assessment of importance of collaboration at the home institution.

#### Additive team tenure

The concept of additive team tenure is the average of team members’ time spent in their team [23–26]. In our survey, participants were asked to provide the length of time they had collaborated with each member of the pilot team, and each participant’s self-reported additive team tenure was calculated as the average of their report of the number of years they have collaborated with each of their pilot team members.

#### Collective team tenure

Collective team tenure is defined as the amount of time that all team members have spent together, also known as the team age [26–28]. Participants’ self-reported collective team tenure was calculated as the minimum of their report of the number of years they have collaborated with each of their team members, that is, collective team tenure becomes 0 when a new member joins the team.

#### Dispersion in team tenure

Dispersion in team tenure describes the variability in the length of time that team members have worked with each other [26,29–31]. Others have suggested that the variability in team tenure can promote workgroup diversity and add a greater variety of knowledge and experiences to the team [29]. From our survey, we calculated each participant’s self-reported dispersion in team tenure as the standard deviation of the number of years they have collaborated with each of their pilot team members.

### Quantitative Outcomes

Quantitative outcomes were the number and type of scholarly products (publications, grant proposals, and grants awarded) that resulted in relation to the initial pilot award. Publications related to the pilot award were determined by searching Scopus and PubMed for manuscripts and abstracts published after the pilot approval date that include at least one member of the pilot team as co-author and contain at least one title keyword match with the pilot award title. When relatedness with the pilot award was unclear based on the publication title, abstracts were analyzed or pilot team members were consulted to determine relatedness between the published work and the pilot award. Grant proposals submitted that are related to the pilot award were determined by searching the university database for submitted proposals meeting the following criteria: (1) submitted after the pilot approval date, (2) principal investigator of the submission is a member of the pilot award team, and (3) submission is related to the pilot award as determined via title keyword match, abstract review, and pilot awardee assessment. Submissions for non-competing renewals, internal awards, and center funding mechanisms were excluded, and some types of submissions, such as Veteran’s Affairs proposals, are not present in the database and thus were searched manually. Subsequent grants awarded that are related to the pilot award were determined using the same process as was used for identifying related grant proposals, except that awarded grants were searched rather than submitted proposals.

The primary outcome of interest is a summary measure of total scholarly products (publications, grant proposals submitted, and grants awarded) resulting from each team. Because the goal of the pilot funding mechanisms was to generate subsequent grant awards, especially R01s, we defined the total scholarly product score by giving more weight to products of more importance and/or more difficulty to attain. Points were assigned for each scholarly product as 1 point for each publication, 2 points for each non-R01 grant proposal submitted, 3 points for each R01 proposal submitted, 4 points for each non-R01 grant awarded, and 5 points for each R01 awarded. Each team’s total scholarly product score is a sum of these points. While primary focus is on this summary measure of total scholarly products, we also performed secondary analyses based on the outcome of whether or not the team received an R01.

### Quantitative Analyses

Team-level independent variables were utilized for examining associations with team-level outcomes. Due to the small number of teams (n = 12), we examined only bivariate, unadjusted analyses for the scholarly product outcomes. To examine associations with the total scholarly product score (primary outcome), graphical analyses were used to assess linearity between each independent variable and the total scholarly product score and to identify the presence of any large influential data points. When no deviations from linearity or large influential points were identified, Pearson’s correlation and 95% confidence intervals were calculated to examine the association with the total scholarly product score. Spearman’s correlation with 95% confidence intervals (calculated via bias-corrected accelerated bootstrap [32,33]) were used otherwise. To examine associations with receipt of a subsequent R01 award (secondary outcome), means and standard deviations were calculated for each independent variable, stratified by whether or not the team received an R01, and Wilcoxon rank sum tests were also performed. Nonparametric Wilcoxon rank sum tests were utilized due to the very small number of teams in each R01 group (*n* = 4 and *n* = 8 that did and did not receive an R01 during follow-up, respectively). P-values were calculated for the primary and secondary analyses with statistical significance considered at *p* < 0.05, but due to the small sample size and exploratory nature of this study, focus is on descriptive statistics and measures of association, not on statistical inference. All quantitative analyses were performed using R version 4.1.2 [34].

### Qualitative Analyses

For the qualitative component of the study, the following primary steps were taken to analyze the textual data elicited in the in-depth interviews. Interviews were recorded and transcribed verbatim using a transcription service, and transcripts were reviewed and verified for accuracy by a member of the research team. Following initial reading of the transcripts, the qualitative team developed a coding scheme for the interview data in NVivo [35]. To ensure robust coding, each interview was independently coded by at least two members of the team. The coded transcripts were synthesized to identify the primary themes in the data using the principles of thematic analysis [36]. In this manuscript, we examine themes related to team interactions and team formation and their relationship to research-related impacts.

## Results

### Quantitative Results

Individual survey respondents were approximately balanced between males (47.8%) and females (52.2%), predominantly White (84.8%), and most had a doctoral degree (91.3%) (Table 1). Respondents spanned all 12 research teams that were granted a pilot award, with 2–8 individual team members (mean = 3.9) responding from each team (Table 2). Across teams, the additive team tenure was, on average, 5.1 years (SD = 2.2), the collective team tenure was, on average, 2.8 years (SD = 1.2), and the dispersion (standard deviation) in team tenure was, on average, 2.5 years (SD = 1.3) (Table 2).

**Table 1.** Demographic information of study participants

	All participants, *n* = 47
Gender	
Female	24 (52.2%)
Male	22 (47.8%)
Race/ethnicity	
American Indian or Alaskan Native	0 (0.0%)
Asian	7 (15.2%)
Black/African American	0 (0.0%)
Hispanic or Latino	0 (0.0%)
Native Hawaiian or Other Pacific Islander	0 (0.0%)
White	39 (84.8%)
Highest degree	
Bachelor’s	2 (4.3%)
Master’s	2 (4.3%)
Doctoral	
MD only	8 (17.4%)
PhD only	26 (56.5%)
MD/PhD	3 (6.5%)
Other (PharmD, DO, DNP, etc.)	5 (10.9%)
Role on team	
Principal investigator	17 (36.2%)
Co-investigator	19 (40.4%)
Professional research staff	5 (10.6%)
Student	1 (2.1%)
Other	5 (10.6%)
General discipline	
Basic sciences (biology, chemistry, etc.)	4 (8.7%)
Behavioral sciences (psychology, counseling, etc.)	6 (13.0%)
Data sciences (biostatistics, informatics, etc.)	4 (8.7%)
Medicine (cardiology, neurology, primary care, etc.)	23 (50.0%)
Nursing	3 (6.5%)
Pharmacy	5 (10.9%)
Public health	1 (2.2%)
Years in discipline	18.8 ± 10.0

MD, Doctor of Medicine; PhD, Doctor of Philosophy; PharmD, Doctor of Pharmacy; DO, Doctor of Osteopathic Medicine; DNP, Doctor of Nursing Practice.

Categorical variables are reported as n (percent). Continuous variables are reported as mean ± standard deviation. Summaries are reported using available data for each variable. The following variables had missing observations: gender (*n* = 1), race/ethnicity (*n* = 1), highest degree (*n* = 1), and discipline (*n* = 1). Disciplines are binned into general categories for ease of reporting, but there is heterogeneity of specific disciplines within the general categories presented in this table.

**Table 2.** Team characteristics and scholarly product outcomes

	All teams, *n* = 12
Number of participants	3.92 ± 1.78
Number of different disciplines	
1	2 (16.7%)
2	6 (50.0%)
3	3 (25.0%)
4	1 (8.3%)
Additive team tenure, years	5.09 ± 2.19
Collective team tenure, years	2.80 ± 1.19
Dispersion (SD) in team tenure, years	2.45 ± 1.34
Number of publications	3.42 ± 2.27
Number of grants submitted	2.25 ± 1.48
Number of grants awarded	1.83 ± 1.11
Number of R01s submitted	
0	8 (66.7%)
1	2 (16.7%)
2	1 (8.3%)
3	1 (8.3%)
Number of R01s awarded	
0	8 (66.7%)
1	2 (16.7%)
2	2 (16.7%)
Total scholarly product score	16.3 ± 9.8
Length of follow-up (months)	52.9 ± 6.7

SD, standard deviation.

Number of different disciplines refers to the number of general disciplines represented on each team using broad categories of basic sciences, behavioral sciences, data sciences, medicine, nursing, pharmacy, and public health; among individuals, there is heterogeneity of specific disciplines within these general categories. Additive team tenure is the average number of years individuals on the team have collaborated with each other. Collective team tenure is minimum number of years all individuals on the team have collaborated with each other, that is, collective team tenure becomes 0 when a new member joins the team. Dispersion in team tenure is the standard deviation of the number of years individuals on the team have collaborated with each other. All team tenure variables are aggregated on the team level as the average of individual team members’ responses. The study period is defined as at the time after each pilot award date through October 2021. R01 submissions and awards are restricted to this study period, so it is possible for there to be more awards than submissions during the study period.

Individuals’ ratings of collaboration history and experience with their pilot team members were generally very favorable (mean satisfaction with team members = 4.42 out of 5, mean team collaboration rating = 4.51 out of 5, and mean quality of team interactions as assessed via the Team Performance Survey = 5.05 out of 6) (Table 3). Individuals’ attitudes about transdisciplinary research in general were also quite favorable (mean = 4.35 out of 5), but the mean rating of importance of collaboration at the home institution was somewhat lower (mean = 3.68 out of 5) (Table 3).

**Table 3.** Collaborative research experiences and attitudes, both with the pilot award project and in general

	Individual responses, *n* = 47	Team-level aggregates, *n* = 12
Satisfaction with pilot award team members	4.42 ± 0.64	4.56 ± 0.36
Team collaboration on pilot award	4.51 ± 0.67	4.70 ± 0.36
Quality of team interactions on pilot award	5.05 ± 1.01	5.28 ± 0.38
Attitude about transdisciplinary research in general	4.35 ± 0.60	4.35 ± 0.60
Assessment of importance of collaboration at home institution	3.68 ± 0.86	3.61 ± 0.55

Each variable is assessed via a 5-point Likert scale except “quality of team interactions on pilot award” which is assessed via a 6-point Likert scale. For all measures, higher numbers represent more positive or favorable views. Team-level summaries of each variable are aggregated as the median of the individual responses among members of each team. All individual-level and aggregated team-level information is summarized as mean ± standard deviation. The following variables had missing observations: satisfaction with pilot award team members (*n* = 2 individuals), assessment of importance of collaboration at home institution (*n* = 1 individual).

**Fig. 1.**
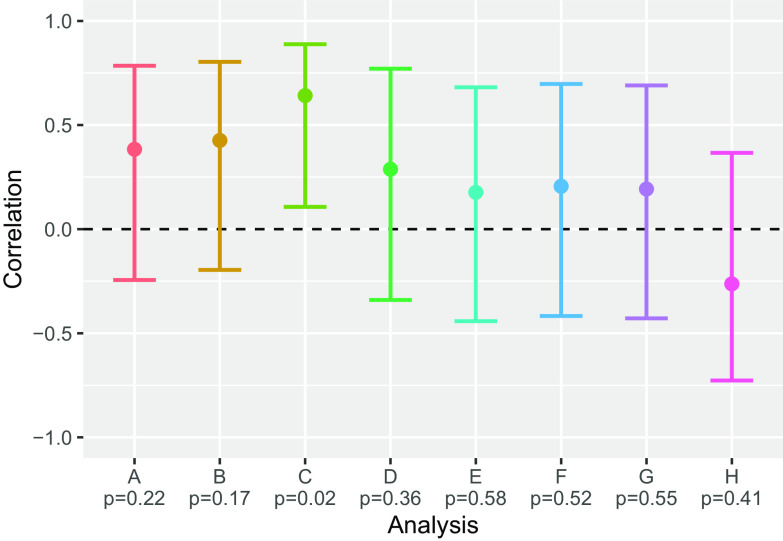
Correlation with total scholarly product score. Points show estimated correlation between total scholarly product score and satisfaction with team members (A), team collaboration (B), quality of team interactions assessed via the Team Performance Survey (C), attitudes about transdisciplinary research in general (D), assessment of importance of collaboration at the home institution (E), additive team tenure (F), collective team tenure (G), and dispersion in team tenure (H). Error bars represent 95% confidence interval for correlation. Pearson’s correlation is utilized for all analyses except (D) which utilizes Spearman’s correlation. Corresponding *p*-values are indicated beneath each analysis. The dotted line represents no correlation between the variables.

Pilot teams produced, on average, 3.42 publications, 2.25 grant proposals, and 1.83 awarded grants during the follow-up period (Table 2). The total scholarly product scores for each team ranged from 1 to 37 with an average of 16.3 (SD = 9.8) (Table 2). The total scholarly product score was positively associated with all three variables representing collaboration within the pilot team, including: satisfaction among team members (*r* = 0.38 [95% CI: −0.24, 0.78]), assessment of team collaboration (*r* = 0.43 [95% CI: −0.20, 0.80]), and quality of team interaction as assessed via the Team Performance Survey (*r* = 0.64 [95% CI: 0.11, 0.89]) (Fig. 1). The association with scores on the Team Performance Survey was statistically significant (*p* = 0.02), suggesting that teams with better quality of interactions (higher scores on the Team Performance Survey) tend to have higher total scholarly product scores. Confidence intervals for these correlations are wide due to the small sample size (*n* = 12 teams), but the magnitude of the effects for the satisfaction among team members and assessment of team collaboration scales suggests a moderate positive association, where teams with higher satisfaction with team members and better team collaboration tended to have higher total scholarly product scores, though these were not statistically significant (*p* = 0.22 and *p* = 0.17, respectively).

One influential point necessitated the use of Spearman’s correlation for analyses involving the attitude about transdisciplinary research in general. Teams’ attitudes about transdisciplinary research in general were positively associated with total scholarly product scores (*r_s_* = 0.29 [95% CI: −0.34, 0.77]), but the association between team members’ overall assessment of importance of collaboration at their home institution and the total scholarly product score was smaller (*r* = 0.18 [95% CI: −0.44, 0.68]) (Fig. 1).

The correlations between additive team tenure and collective team tenure with the total scholarly product score were also positive but small (*r* = 0.21 [95% CI: −0.42, 0.70], *r* = 0.19 [95% CI: −0.43, 0.69], respectively) (Fig. 1). Dispersion in team tenure was negatively associated with the total scholarly product score (*r* = −0.26 [95% CI: −0.73, 0.37]), where teams that had more variability in the length of time that each team member had collaborated with each other tended to have lower total scholarly product scores, though this negative association was somewhat weak (Fig. 1).

Secondary analyses revealed that teams that were awarded a subsequent R01 grant related to the pilot project tended to have higher ratings of team collaboration (received R01, mean ± SD: 4.94 ± 0.07; did not receive R01, mean ± SD: 4.59 ± 0.39) and quality of team interactions as assessed via the Team Performance Survey (received R01, mean ± SD: 5.47 ± 0.43; did not receive R01, mean ± SD: 5.18 ± 0.35), though neither of these differences were statistically significant (*p* = 0.10 and *p* = 0.27, respectively) (Table 4). Satisfaction with pilot award team members, attitudes about transdisciplinary research in general, assessment of importance of collaboration at the home institution, and the three team tenure variables were all not statistically or functionally different between teams that did and did not receive a subsequent R01 (Table 4).

**Table 4.** Associations between receiving an R01 and pilot team collaboration, beliefs, and structure

	Received R01 (*n* = 4)	Did not receive R01 (*n* = 8)	*p*-value
Satisfaction with pilot award team members	4.55 ± 0.43	4.56 ± 0.36	>0.99
Team collaboration on pilot award	4.94 ± 0.07	4.59 ± 0.39	0.10
Quality of team interactions on pilot award	5.47 ± 0.43	5.18 ± 0.35	0.27
Attitude about transdisciplinary research in general	4.11 ± 1.05	4.47 ± 0.21	0.68
Assessment of importance of collaboration at home institution	3.47 ± 0.82	3.69 ± 0.42	0.55
Additive team tenure, years	5.50 ± 2.32	4.89 ± 2.26	0.68
Collective team tenure, years	2.81 ± 1.28	2.79 ± 1.23	0.73
Dispersion (SD) in team tenure, years	2.48 ± 1.35	2.44 ± 1.43	0.93

Variables are team-level and summarized by mean ± standard deviation, stratified by whether or not the team received a subsequent R01 grant related to the pilot project. P-values are from Wilcoxon rank sum tests. Satisfaction with pilot award team members, team collaboration on pilot award, attitudes about transdisciplinary research in general, and assessment of importance of collaboration at the home institution are assessed via 5-point Likert scale where higher numbers represent more positive or favorable views. Quality of team interactions is assessed via the Team Performance Survey, a 6-point Likert scale where higher numbers represent more positive or favorable views. Additive team tenure is the average number of years individuals on the team have collaborated with each other. Collective team tenure is minimum number of years all individuals on the team have collaborated with each other, that is, collective team tenure becomes 0 when a new member joins the team. Dispersion in team tenure is the standard deviation of the number of years individuals on the team have collaborated with each other. All team tenure variables are aggregated on the team level as the average of individual team members’ responses.

### Qualitative Results

The in-depth interview sample was balanced by gender (six females, six males), career stage (five were early career at the time of the award, seven were senior investigators), and training (six were PhDs; six were MD, clinician scientists). Four individuals mentioned being new faculty at the institution at the time of award.

Consistent with the survey results on team tenure, most interviewees noted that their pilot teams consisted of a core of established collaborators, with new members added for specific disciplinary expertise when gaps were identified or in response to evolving scientific questions. All were by design multidisciplinary teams, and typically between four and five disciplinary areas were represented on the awarded teams.

#### Team formation and interaction

A primary theme that emerged from the interviews related to team formation was the importance of the MVP/VI^2^P pilot projects in providing a project-based mechanism to formalize or cement collaborative relationships among the team members. This was especially salient for early career faculty and those new to the institution, who explicitly mentioned these award mechanisms providing the vehicle to solidify emerging collaborative relationships, foster relationships with new departments, and/or advance the work of the team, for example, by moving preliminary research into a clinical trial phase which the team had not previously undertaken. One early career investigator noted that:

“I came to [this institution] with an interest of being a clinician scientist but did not come with funding or protected time. So, I have had to develop these relationships and then apply for these grants to try to build that career trajectory. I think that the VI^2^P was my first sort of larger grant where I was a PI and it was a good learning experience…. It helped accelerate, kind of got my name out there, and I think that’s helped within the department to give me some stature as a researcher.”

Study teams engaged in a range of different processes to facilitate communication and optimize time and progress on their pilot studies. All teams commented on the importance of regular structured communication among the team members for team building and achieving progress but also highlighted meeting efficiency as a priority for the teams. One senior investigator noted that the team’s dynamic of shared responsibility and internal pressure for progress was key to propelling the work and making the meetings productive.

A key theme arose around setting clear expectations and establishing shared goals as key elements of effective team building. This theme was especially prevalent among the more senior investigators we interviewed, though it was also mentioned by one early career PI as well. Strategies tended to involve goal-directed communication as a way of organizing the team’s work, and establishing a shared vision for the team’s work, with all members understanding their roles and seeing the benefits of participation.

“I think a really important first step is bringing the team together, sharing their expectations, sharing their vision, and getting on the same page. Actually establishing the baseline and then developing the framework of what type of communication is going to work best for your group.”

“So the first is just establishing before a project even starts, what are the benefits for each of the team members, right? So what are their end goals and how does this project help move things forward for them? What we don't want is trying to establish this collaborative relationship, but then it turns into busy work for this other person, or there’s an undue expectation of being involved in something that isn't necessarily their specialty area. So we've really gone out of our way to share that information just openly among all the team members.”

Relatedly, teams identified a need for specific training to optimize teamwork and increase team performance as key priorities. These ideas ranged in scope from training with tools and processes to enhance effectiveness of existing, mature scientific teams, to specific training in protocol development and writing for early-stage clinician-scientists. For example, one participant noted that:

“We all could benefit from some more training in team science… I think this project worked because we were, the core of us, were already working together. So we had a lot of the tools that we needed to be able to do the project. You know, we'd already done clinical trials, we've enrolled patients and so, as we think about how we could have made it even more effective, I think employing some of some more team science tools could have helped the group.”

#### Funding outcomes

The investigators we interviewed were keenly aware that the MVP and VI^2^P pilot mechanisms were designed to position the awarded teams for successful extramural funding submissions, and particularly for progression to R01-level submissions. Four teams were successful on the R01 trajectory, including clinician scientists who were seeking their first R01 funding, who noted the critical role of pilot support and mentoring resources as key elements of success:

“We really needed to use these funds and this time to prepare us as we're moving toward an R01 application, to keep that goal before us. I think that that common goal of a large grant that really gives us the resources, the time, and the recognition to be able to adequately do this work was critical. And I think that goal reinforced to us as well as the timelines for, okay, when do we need to have this R01 application completed?”

Interestingly, several teams experienced significant challenges in the course of their planned scientific activities, and made nimble adjustments to adapt their scientific inquiry, that led to unforeseen but successful alterations in the research trajectory:

“The MVP grant, for sure, had a strong influence on the formation of what is now our very large collaboration. It wasn't part of the funded project of the MVP… but the data from that has already been used to fuel three extramural funding applications.”

Other investigators we interviewed echoed this notion of altered trajectories or “off-shoot” projects that, although they couldn't be traced directly as the next step from the original pilot, were influenced by a key finding or new collaboration that advanced in a different direction:

“We did identify, and we've published on, a novel pathway that emerged from the work. I will share that this was a real challenge to do this project and really to take it to the next step. We've gotten funding for shoot-off projects. I think we now have three federally funded grants… but they're all about the basic science and using pre-clinical models. Neither of us have really been able to get the translational aspect funded. It’s just such a big task to take it to the next step. Somebody with a lot more resources to invest has to get interested in it to really move it to the next phase.”

## Discussion

In this mixed-methods study of multidisciplinary research teams, three collaborative factors that we studied had moderate or strong positive associations with the total number of scholarly products produced by the team: satisfaction with team members, team collaboration, and quality of team interactions. Self-report of team collaboration on the project was also somewhat higher on teams that subsequently obtained an R01 award compared to those that did not. Attitudes about transdisciplinary research in general, assessment of the importance of collaboration at the home institution, and factors related to the duration of collaboration with team members had weak associations with scholarly product outcomes.

The number of research teams in this study was small: survey responses were collected for 47 individuals, but these participants were from only 12 different teams. The small number of teams limits the amount of quantitative information available about team-based outcomes and thus limits our ability to make inference beyond our sample. The nonparametric tests used for inference in this study can also have lower power than their parametric counterparts. Although all but one of the aforementioned associations did not meet the predetermined threshold for statistical significance, the effect sizes still warrant future study in a larger number of research teams. Additionally, the qualitative results from this study support the notion that team dynamics among research collaborators from multiple disciplines were key in facilitating the achievement of scholarly products.

Overall, participants in our study had very favorable ratings of the interactions among their pilot team members and their general views about transdisciplinary research outside of the interactions on the pilot team. Because the self-reports of collaborative experiences were predominantly very positive, there was a ceiling effect with the Likert scales used to assess these experiences – individuals’ ratings of their collaborative experiences were very high, and there was low variance in the scores. While this feature of our quantitative data is positive for the teams themselves, it does reduce the ability of our study to identify associations between these collaborative variables and the outcomes of interest. There is also a possibility that our survey was subject to response bias if researchers with more positive experiences or more favorable views about collaborative research in general were more likely to respond. Collecting data about satisfaction with each team member precluded survey anonymity and possibly deterred respondents from reporting negative experiences with their collaborators. Future work should aim to recruit researchers with a more diverse range of collaborative experiences to examine associations with scholarly product outcomes more effectively.

The quantitative portion of our study focused on scholarly outcomes to evaluate the success of research teams because these metrics are objective, easy to measure, and commonly used in practice to evaluate research teams [11]. However, a fixed follow-up period was utilized for this study, and teams may have continued to produce scholarly outcomes after the follow-up period. In the absence of an institutional database for tracking subsequent grant proposals and awards, individual self-report could be used to identify scholarly products for research teams or individual researchers in future work. For this exploratory study, a simple weighting scheme was used to give more weight to R01 submissions and awards because these were the specific target for return-on-investment of the pilot award programs being studied, but other weighting schemes could be utilized to give differential weight to the scholarly products. Also, simple scholarly metrics such as publication counts do not consider the impact of the work on clinical care or in communities. Others have discussed the challenge of quantifying the success of collaborative research teams and have suggested several possible solutions [11]. One such solution to quantify the impact of translational research is the Translational Science Benefits Model (TSBM) which examines benefits to clinical care, public health, the economy, and/or policy [37]. Future directions from this study will explore associations between team dynamics and TSBM outcomes. Future studies may also wish to examine how collaborative team dynamics or pilot award experiences impact scholarly outcomes achieved by individual researchers in addition to the scholarly outcomes achieved on a team level.

Although some limitations were noted with the quantitative outcomes in this study, a strength of the study is the mixed methods approach. Results from the qualitative portion of this study highlighted additional positive outcomes from the multidisciplinary teams that were not captured in the quantitative survey results. One such outcome is the potential for career development and acceleration, particularly for early career investigators who collaborated with more senior investigators. Future work could capture more information about this outcome by utilizing scales to assess career development and/or mentorship with research collaborators.

The qualitative results of this study also shed light on strategies that could improve collaborative, transdisciplinary work. Teams that were successful collaboratively noted that the early stages of team development were particularly important for establishing shared goals and communication strategies to foster effective collaborative work. Many participants in this study also indicated that receiving specific training in team science would have been beneficial. Others have worked to develop team science-related training/interventions and recommendations for transdisciplinary collaborative work that can and should be utilized to meet this need [12,38–40].

The results of this study may be generalizable to multidisciplinary research teams at other large research universities or academic medical centers, especially those that are funded by pilot awards that specifically incentivize team science. The survey items utilized for this study were derived from scales previously crafted for the purpose of evaluating work from Clinical and Translational Science Award programs or other similar collaborative programs, so the survey questions are not specific to our institution. One exception to the generalizability of this study is that analyses utilizing the variable, “assessment of importance of collaboration at home institution,” may not generalizable beyond our institution, as respondents are reacting to their opinion of the specific environment at this institution.

In conclusion, our study demonstrates the positive impact of pilot awards aimed to foster multidisciplinary collaboration at our institution. Quantitatively, our study shows preliminary evidence that satisfaction with team members, team collaboration, and quality of team interactions are all positively associated with the creation of scholarly products, and these effects warrant future study in a larger number of multidisciplinary research teams. Qualitative results support these associations and also add several benefits of initiating multidisciplinary teams including career advancement opportunities for early career investigators.
